# Ionizing radiation induced degradation of diuron in dilute aqueous solution

**DOI:** 10.1186/s13065-015-0097-0

**Published:** 2015-04-18

**Authors:** Krisztina Kovács, Shijun He, Viktoria Mile, Tamás Csay, Erzsébet Takács, László Wojnárovits

**Affiliations:** Institute for Energy Security and Environmental Safety, Centre for Energy Research, Hungarian Academy of Sciences, Budapest, Hungary; Institute of Nuclear and New Energy Technology (INET), Tsinghua University, Beijing, 100084 China; Faculty of Light Industry and Environmental Engineering, Obuda-University, Budapest, Hungary

**Keywords:** Diuron, Hydroxyl radical, Degradation, Advanced oxidation processes, Irradiation technology

## Abstract

**Background:**

Cutting edge technologies based on Advanced Oxidation Processes (AOP) are under development for the elimination of highly persistent organic molecules (like pesticides) from water matrices. Among them, ionizing radiation treatment represents a promising technology that requires no additives and can be easily adapted to an industrial scale. In these processes several reactive species are produced, mainly powerful oxidizing radicals inducing the degradation. This paper investigates the reactions taking place in dilute aqueous solutions of a hazardous pollutant (diuron) during irradiation.

**Results:**

Irradiation of aqueous diuron solutions resulted in effective degradation of the solute mainly due to the reactions of hydroxyl radicals formed in water radiolysis. Hydroxyl radical reacts with diuron with a second order rate constant of (5.8 ± 0.3) × 10^9^ mol^−1^ dm^3^ s^−1^. The main reaction is addition to the ring forming hydroxycyclohexadienyl radical. 30 − 50% of hydroxyl radical reactions induce dechlorination. Reactions with the methyl groups or with the α-amino group have low contribution to the transformation. The presence of dissolved oxygen enhances the rate of degradation; one hydroxyl radical on average induces five-electron oxidations. The high oxidation rate is attributed to the reaction of some of the primarily formed organic radicals with dissolved O_2_ and the subsequent reactions of the peroxy radicals.

**Conclusion:**

The presence of dissolved oxygen is highly important to achieve efficient ionizing radiation induced degradation of diuron in dilute aqueous solution.

## Background

Diuron, a phenylurea herbicide, has been used worldwide in agriculture to control broadleaf and grassy weeds in cereals and other crops for more than 50 years. Diuron is highly persistent in the aquatic environment due to its photochemical stability. As a consequence, it has been detected in wastewater effluents and in surface waters at low concentrations up to the μg dm^−3^ level. As a potentially carcinogenic substance, its occurrence in such reservoirs poses serious threat to human health and it is also toxic to microorganisms. The European Water Framework Directive classifies diuron as one of the priority substances, being a hazardous pollutant [1]. Diuron is also included in the drinking water contaminant candidate list of the U.S. Environmental Protection Agency [2], to enforce further research and data collection.

Advanced Oxidation Processes (AOP) are suggested for the elimination of persistent organic compounds from water matrices. The radical induced degradation of diuron has been investigated formerly using several AOP [3-7]. In these experiments, with the exception of direct photodegradation, hydroxyl radical was the main oxidizing agent. Mazellier and Sulzberger [8] observed only one product in heterogenous photo-Fenton system (3-(3,4-dichlorophenyl)-1-formyl-1-methylurea), indicating ^•^OH attack on the methyl group. In other experiments using ozonation or photocatalytic oxidation, the ^•^OH attack took place on the aromatic ring leading to hydroxylated or dechlorinated products.

The ionizing radiation treatment is one of the AOP. In establishing reaction mechanisms radiation chemistry provides the advantage of investigating the radical intermediates by electron pulse radiolysis (their yields can be easily calculated) and additionally achieving the final products by utilizing ^60^Co γ-irradiation. Radiation technology can be applied on an industrial scale [9]. The aim of the present study is to investigate both the intermediates and the final products of diuron degradation induced by water radiolysis techniques. In order to promote the practical application we also determined sum parameters important in wastewater analysis: chemical oxygen demand (COD), total organic carbon (TOC) and total nitrogen content (TN). To the best of our knowledge, there is only one publication reporting the radiation induced degradation of diuron [10]. In a previous paper we have already reported on the degradation of fenuron [11].

## Materials and methods

### Chemicals and equipment

Diuron and other chemicals were purchased from Spectrum-3D or Carlo Erba and used without further purification. HPLC grade eluents for liquid chromatographic analysis were obtained from Fischer Chemicals. All solutions were prepared at their natural pH. The absorption spectra of the irradiated samples were recorded on a JASCO 550 UV-Vis spectrophotometer in a 1 cm cell. Appropriate dilutions were applied in order to stay within the linear range of the Lambert-Beer relation. An Agilent Technologies 6410 Triple Quad HPLC-MS/MS system was used for final product identification. Liquid chromatographic separations were carried out on a Phenomenex Kinetex Phenyl-Hexyl column (100 mm × 2.1 mm × 2.6 μm) using isocratic elution mode at a flow rate of 0.25 cm^3^ min^−1^. The mobile phase composed of 45% methanol and 55% water. The concentration of adsorbable organic halides (AOX) and free chloride ions were monitored by AOX equipment and perfectION^TM^ Combination Chloride Electrode, respectively. COD measurements were performed with a Behrotest TRS 200 COD system. TOC and TN were measured using Shimadzu TOC-VCSN equipment.

### Pulse radiolysis and steady-state gamma irradiation

Pulse radiolysis was performed using 800 ns pulses of a linear accelerator, and an optical detection system with a cell having 1 cm light path length [12]. Pulse dosimetry was carried out in air-saturated, 1 × 10^−2^ mol dm^−3^ KSCN solutions by monitoring the absorbance of (SCN)_2_^−•^ at *λ*_max_ 480 nm. The absorbed doses per pulse were 20 Gy. A ^60^Co gamma facility with 1.8 PBq activity was used for steady-state gamma irradiation. The dose rate was measured to be ~10 kGy h^−1^ using ethanol-monochlorobenzene (ECB) dosimetry.

In the radiolysis of water, hydrated electron (e_aq_^−^), hydrogen atom (H^•^) and hydroxyl radical (^•^OH) form as reactive intermediates as shown in Eq. (1) [13,14].

1 $$ {\mathrm{H}}_2\mathrm{O}\ {\mathrm{e}}_{\mathrm{aq}}\rightsquigarrow (0.28) + \kern0.5em {\mathrm{H}}^{\bullet }(0.07) + {\kern0.5em }^{\bullet}\mathrm{O}\mathrm{H}(0.28) $$

2 $$ {{\mathrm{e}}_{\mathrm{aq}}}^{\hbox{-} }+\kern0.5em {\mathrm{N}}_2\mathrm{O}\kern0.5em +\kern0.5em {\mathrm{H}}_2\mathrm{O}\kern0.5em \to {\mathrm{OH}}^{\hbox{-}}\kern0.5em +{\kern0.5em }^{\bullet}\mathrm{O}\mathrm{H} + \kern0.5em {\mathrm{N}}_2\kern0.5em \left(k\kern0.5em =\kern0.5em 9.1\times {10}^9{\mathrm{mol}}^{\hbox{-} 1}{\mathrm{dm}}^3{\mathrm{s}}^{\hbox{-} 1}\right) $$

3 $$ {}^{\bullet}\mathrm{O}\mathrm{H} + {\left({\mathrm{CH}}_3\right)}_3\mathrm{C}\mathrm{O}\mathrm{H}\kern0.5em {\to}^{\bullet }{\mathrm{CH}}_2{\left({\mathrm{CH}}_3\right)}_2\mathrm{C}\mathrm{O}\mathrm{H} + {\mathrm{H}}_2\mathrm{O}\kern0.5em \left(k = 6\times {10}^8{\mathrm{mol}}^{\hbox{-} 1}{\mathrm{dm}}^3{\mathrm{s}}^{\hbox{-} 1}\right) $$

4 $$ {{\mathrm{e}}_{\mathrm{aq}}}^{\hbox{-} } + {\mathrm{O}}_2\kern0.5em \to {{\mathrm{O}}_2}^{\hbox{-} \bullet}\kern0.5em \left(k\kern0.5em  = 1.9\times {10}^{10}{\mathrm{mol}}^{\hbox{-} 1}{\mathrm{dm}}^3{\mathrm{s}}^{\hbox{-} 1}\right) $$

5 $$ {\mathrm{H}}^{\bullet } + {\mathrm{O}}_2\kern0.5em \to {{\mathrm{H}\mathrm{O}}_2}^{\bullet}\kern0.5em \left(k\kern0.5em  = 2.1\times {10}^{10}{\mathrm{mol}}^{\hbox{-} 1}{\mathrm{dm}}^3{\mathrm{s}}^{\hbox{-} 1}\right) $$

6 $$ {{\mathrm{H}\mathrm{CO}}_2}^{\hbox{-}}\kern0.5em +{\kern0.5em }^{\bullet}\mathrm{O}\mathrm{H}\kern0.5em \to \kern0.5em {{\mathrm{CO}}_2}^{\hbox{-} \bullet}\kern0.5em +\kern0.5em {\mathrm{H}}_2\mathrm{O}\ \left(k\kern0.5em  = 3\times {10}^9{\mathrm{mol}}^{\hbox{-} 1}{\mathrm{dm}}^3{\mathrm{s}}^{\hbox{-} 1}\right) $$

7 $$ {{\mathrm{CO}}_2}^{\hbox{-} \bullet } + {\mathrm{O}}_2\kern0.5em \to \kern0.5em {{\mathrm{O}}_2}^{\hbox{-} \bullet}\kern0.5em  + {\mathrm{CO}}_2\kern0.5em \left(k\kern0.5em  = 4.2\times {10}^9{\mathrm{mol}}^{\hbox{-} 1}{\mathrm{dm}}^3{\mathrm{s}}^{\hbox{-} 1}\right) $$

In Eq. (1) the numbers in parentheses are the radiation chemical yields (*G*-values) in μmol J^−1^. Standard radiation chemical techniques were applied in the experiments in order to observe the individual reactions of the intermediates. The ^•^OH reactions were followed in N_2_O-saturated solutions to convert e_aq_^−^ to ^•^OH in Reaction (2). Reactions between e_aq_^−^ and diuron were investigated in N_2_-saturated solutions containing 5 vol. % *tert*-butanol to convert ^•^OH to the less reactive ^•^CH_2_(CH_3_)_2_COH in Reaction (3). When the solution was purged with N_2_ in the absence of *tert*-butanol, all the three primary species reacted with diuron. In the presence of dissolved O_2_ (air or O_2_ saturated solutions), e_aq_^−^ and H^•^ are converted to superoxide radical anion/perhydroxyl radical pair in Reactions ((4) and (5)). Hence, in these solutions ^•^OH as well as the O_2_^−•^/HO_2_^•^ pair induce the transformations (p*K*_a_ (O_2_^−•^/HO_2_^•^) = 4.8). Experiments were also made in O_2_ saturated solutions containing Na formate (0.05 mol dm^−3^). In such solutions (in addition to e_aq_^−^ and H^•^) ^•^OH is transformed to O_2_^−•^/HO_2_^•^ in Reactions (6) and (7).

In most of the experiments ~1 × 10^−4^ mol dm^−3^ diuron solutions were investigated, they were saturated with appropriate gases (N_2_O, N_2_, O_2_ or air) before irradiation.

## Results

### Pulse radiolysis

**Figure 1:**
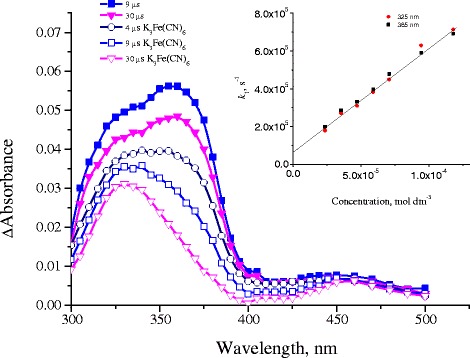
Transient absorption spectra in N_2_O saturated, 1 × 10^−4^ mol dm^−3^ diuron solutions. Closed symbols in the absence, open symbols in the presence of 1 × 10^−4^ mol dm^−3^ K_3_Fe(CN)_6_, dose/pulse 20 Gy. Inset: concentration dependence of pseudo-first-order rate constant of absorbance build-up at 325 and 365 nm.

In the transient absorption spectra taken on samples investigating the ^•^OH reaction (N_2_O saturated), three absorption bands were observed: two shorter wavelength bands peaking at ~330 nm and ~360 nm and a longer wavelength band with maximum around 460 nm (Figure 1). The build-up of these three bands was similar. In separate experiments we added K_3_Fe(CN)_6_ to the solution with equal concentration as diuron. In these solutions the ~360 nm band had smaller intensity and it decayed quickly. The decay of the ~460 nm band took place on the ms time scale.

In general, ^•^OH is a highly reactive electrophilic radical, in its reaction with diuron, however, direct oxidation (electron transfer) hardly occurs [5]: the basic reactions are addition to the ring and H-abstraction from one of the CH_3_ groups or from the N-H group. The absorption band at ~360 nm is typical for the hydroxycyclohexadienyl radicals: e.g. for the chloroanilines the maxima of the bands are around 360 nm [15]. For fenuron the maximum is at 350 nm [11]. Therefore, we assign the ~360 nm band to hydroxycyclohexadienyl radical isomers. Ring-hydroxylated products identified by HPLC-MS/MS technique provide further confirmation. In addition, this identification is in agreement with the fast decrease of this absorption band in the presence of K_3_Fe(CN)_6_. This additive is known to oxidize cyclohexadienyl type radicals to phenols in fast reactions.

The longer wavelength band at *λ*_max_ ~460 nm (and partly the band at 330 nm) may belong to (oxygen centred) phenoxyl radicals or to (nitrogen centred) aminyl radical. As it will be discussed later, phenoxyl radical may form after release of HCl from the ^•^OH adducts. In the case of hydroxyanilines the *λ*_max_ of phenoxyl radicals are around 440 nm [15]. Aminyl radical can form in H-abstraction from N-H group. These radicals are observed in the reaction of SO_4_^•−^ with phenylureas and also in photoionization experiments. In the case of diuron *λ*_max_ ≈ 450 nm and *ε*_max_ ≈ 2000 mol^−1^ dm^3^ cm^−1^ are suggested [3]. Based on the absorption spectra we cannot make a clear assignment for the longer wavelength band. Based on end product experiments (phenol formation) this absorbance can be attributed to phenoxyl radical. Considering the band intensity we can also safely say that H-abstraction from N-H is less important than the other reactions of ^•^OH.

The radical that forms in H-abstraction from a CH_3_ group is expected to exhibit UV absorbance below 300 nm. However, under our circumstances it is invisible since below 300 nm the absorbance of diuron disturbs observing the transient absorbance.

The rate constant of the diuron + ^•^OH reaction was determined by varying the diuron concentration and measuring the build-up of radical absorbance. The slope of the pseudo-first-order rate constant *versus* diuron concentration plot suggests a second order rate constant of (5.8 ± 0.3) × 10^9^ mol^−1^ dm^3^ s^−1^ (Figure 1, Inset). This value is in agreement with the rate constants published previously: their average is calculated to be 6.0 × 10^9^ mol^−1^ dm^3^ s^−1^ [16].

In pulse radiolysis experiments with N_2_-saturated solution containing *tert*-butanol (e_aq_^−^ reaction) low transient absorbance was obtained in the 300 − 400 nm range (not shown) with maximum at ~350 nm. Based on the hydrated electron absorbance decay at 600 nm, we calculated a rate constant of 1.0 × 10^10^ mol^−1^ dm^3^ s^−1^ for the e_aq_^−^ + diuron reaction. Canle Lopez et al. [3] measured (9.4 ± 0.6) × 10^9^ mol^−1^ dm^3^ s^−1^ previously.

### Steady-state gamma radiolysis

**Figure 2:**
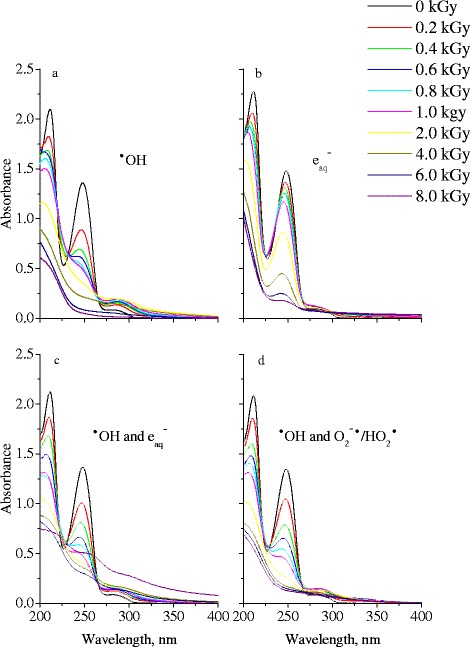
Absorption spectra of 1.6 × 10^−4^ mol dm^−3^ diuron solution. Irradiation with 0 − 8 kGy doses, the spectra were obtained in ^•^OH **(a)**, e_aq_^−^ **(b)**, ^•^OH + e_aq_^−^ **(c)** and in ^•^OH + O_2_^−•^/HO_2_^•^ **(d)** reactions.

The degradation of diuron in gamma radiolysis was followed by UV-Vis spectroscopy and by COD, TOC and TN measurements. Product analysis was also performed using HPLC-MS/MS technique. The band between 230 and 270 nm in the UV spectrum is assigned to the characteristic π → π* transition of the aromatic ring, *λ*_max_ is at 248 nm with *ε*_max_ of 17000 mol^−1^ dm^3^ cm^−1^. The absorbance decreased faster when ^•^OH was the reacting species (Figure 2a) than in e_aq_^−^ reaction (Figure 2b). Moreover, in ^•^OH reaction at ~300 nm increase in absorbance was observed, this build-up was absent in e_aq_^−^ reaction. In both ^•^OH and e_aq_^−^ reactions the absorption band slightly shifted to shorter wavelength. This shift may reflect dehalogenation. *λ*_max_ for the monohalogenated monuron and the non-halogenated fenuron are at 245 and 238 nm (*ε*_max_ 15377 and 13104 mol^−1^ dm^3^ cm^−1^, respectively).

**Figure 3:**
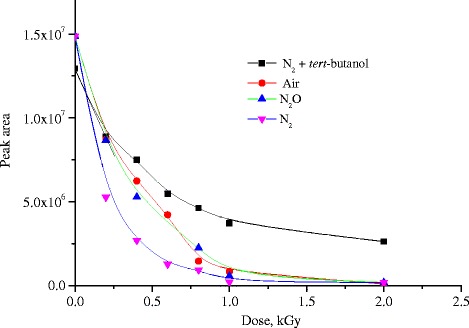
Integrated diuron peaks obtained after HPLC-MS/MS analysis (optical detection). 1 × 10^−4^ mol dm^−3^ solution was irradiated under different conditions indicated in the Figure.

As the integrated chromatographic peak areas (diode array detection) show in Figure 3, at a dose of 1 kGy the majority of diuron degraded in N_2_O (^•^OH), N_2_ (^•^OH + e_aq_^−^) and air saturated (^•^OH + O_2_^−•^/HO_2_^•^) solutions, only a few percent of the intact molecules remained. In N_2_ saturated *tert*-butanol containing solutions (e_aq_^−^), however, the degradation was much slower; the initial yield was ~0.15 μmol J^−1^. In air and N_2_O saturated solutions the values were estimated to be ~0.2 μmol J^−1^ and ~0.3 μmol J^−1^, respectively.

### Identification of by-products by HPLC-MS/MS

**Figure 4:**
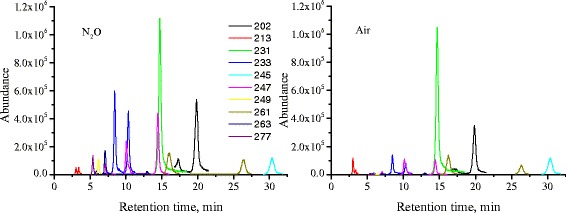
Chromatogram of 1 × 10^−4^ mol dm^−3^ diuron solutions. Irradiation with 0.5 kGy dose in N_2_O or air atmosphere. The peak of each selected ion is depicted with different colour and its m/z value is indicated.

A large number of products were observed on the chromatograms, their composition changed gradually as a function of dose. The distribution was different in air- and N_2_O saturated solutions (Figure 4). At 0.5 kGy dose in both solutions about 50-70% of the starting molecules transformed to products: 14 different compounds were separated. The large variation is partly due to the possible presence of isomers and also to the thermal instability and decomposition of some of the products. Diuron eluted at 14.34 min and in negative ionization mode had a parent ion with m/z 231. Two compounds with m/z 247 eluted earlier than diuron at 6.89 and 14.13 min, respectively. We identified these molecules as ring hydroxylated derivatives of diuron. The ion at m/z 247 had an intensive fragment with m/z 202. This m/z 202 ion appeared also for the ring hydroxylated products with modified -N(CH_3_)_2_ unit: with one CH_3_ group missing (parent ion at m/z 233, 8.37 and 10.12 min), and hydroxylated molecules with one CH_3_ oxidized to –CHO (m/z 261, 16.31 and 25.89 min). In the case of two products at 16.93 and 19.50 min no ion with higher m/z than 202 appeared, indicating a complete ionic degradation of the parent ion in the mass spectrometer. In the case of molecules with one Cl only (parent ion m/z 213, 2.19 and 2.32 min), m/z 202 was not present and another fragment at m/z 168 indicated a chlorine loss (m/z 202-Cl). In the case of ring dihydroxylated molecule (parent ion at m/z 263, 7.01 min) the MS/MS spectra revealed a fragment at m/z 218 (m/z 202 + OH), which contains one more hydroxyl group compared to m/z 202. The product with parent ion m/z 249 at 6.08 min was identified as ring dihydroxylated molecule with one methyl group missing, while the one with m/z 277 at 5.31 min as ring dihydroxylated product with one methyl group oxidized to -CHO. Fragments with m/z 202, m/z 218 (m/z 202 + OH) and m/z 168 (m/z 202-Cl) are characteristic for ring hydroxylated molecules. They form isocyanate ion in the collision chamber, e.g. in the case of diuron: ^−^O(Cl_2_)C_6_H_2_NHCON(CH_3_)_2_ → ^−^O(Cl_2_)C_6_H_2_N=C=O + HN(CH_3_)_2_. Similar reaction (isocyanate ion formation) has also been observed in the thermal degradation of diuron [6] and during the mass spectrometric analysis of irradiated fenuron samples [11].

In the case of the molecule with elution time 29.64 min at m/z 245, the aromatic ring remained intact and one of the methyl groups was oxidized to -CHO.

The products reveal that ^•^OH attack takes place both on the ring and also on the methyl groups attached to the terminal N atom. Most of these products were observed also in previous studies where hydroxyl radicals were the initiating reactants [4,7,10]. According to Tahmasseb et al. [4] “The disubstitution of the phenyl ring by chlorine atoms reduces its susceptibility towards any electrophylic and/or radical attack which in counterpart makes the attack on the urea N-terminus group more competitive.” However, our results clearly show the predominance of ring hydroxylation reactions for diuron similarly to the reactions with fenuron [11].

Figure 4 suggests that the rate of hydroxylated product formation is higher in N_2_O-saturated solution than under aerated condition. This difference may be due to the higher yield of hydroxyl radicals in the former solution than in the latter (0.56 and 0.28 μmol J^−1^) and also to the reaction of the dissolved O_2_ with the organic radicals in aerated solution, opening up by that new reaction pathways at the expense of hydroxylated product formation (see later). Most of the products had maximum concentrations between 0.3 and 0.7 kGy dose. At 1 kGy dose strong decrease in product concentrations was observed.

### Chloride release and AOX

**Figure 5:**
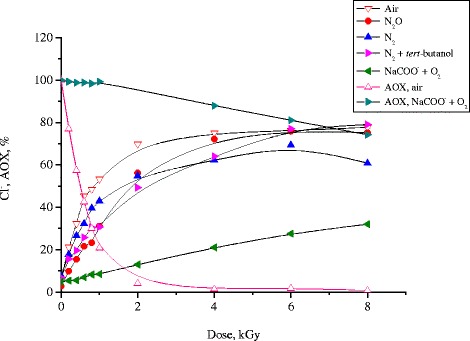
Chloride release (Cl^−^) and the adsorbable organic chloride (AOX). Chloride release was measured in different solutions (indicated in the Figure) AOX was measured in air and O_2_ saturated (Na-formate added) solution (diuron 1.1 × 10^−4^ mol dm^−3^).

Chloride release is an important indicator for the degradation of chlorinated molecules. It facilitates the understanding of the attack of reactive radicals on special sites of the molecule studied. Chloride release was observed under all conditions investigated (Figure 5), in the reactions of ^•^OH, e_aq_^−^, ^•^OH + e_aq_^−^, and ^•^OH + O_2_^−•^/HO_2_. The percentages in the figure are calculated by taking into account both of the chlorine atoms in diuron. The initial (low-dose) yields for air saturated (^•^OH + O_2_^−•^/HO_2_), N_2_ saturated (e_aq_^−^ + ^•^OH), N_2_ saturated containing *tert*-butanol (e_aq_^−^) and N_2_O saturated (^•^OH) solutions are ~0.15, ~0.1, ~0.08 and ~0.07 μmol J^−1^. In O_2_-free solutions about 15 − 30% of ^•^OH attack on diuron yields chloride ion, while this value is about 60% in solutions containing dissolved O_2_. The efficiency of Cl^−^ release for e_aq_^−^ reaction is about 25%.

We investigated the question whether the O_2_^−•^/HO_2_ pair directly induces chloride release. In order to examine the possibility of O_2_^−•^/HO_2_ reaction with diuron, measurements were conducted in O_2_ saturated Na formate containing solutions. In such solutions all the three reactive intermediates of water radiolysis transform to the O_2_^−•^/HO_2_ pair in Reactions (4) − (7). In solutions with Na formate the absorbance slightly decreased with increasing absorbed dose and the maximum slightly shifted to shorter wavelengths (not shown). However, the effect was small and was observable only at higher doses. As Figure 5 shows in Na formate solutions both the yield of chloride release and decrease of AOX were small at low doses.

### Removal of COD, TOC and TN

**Figure 6:**
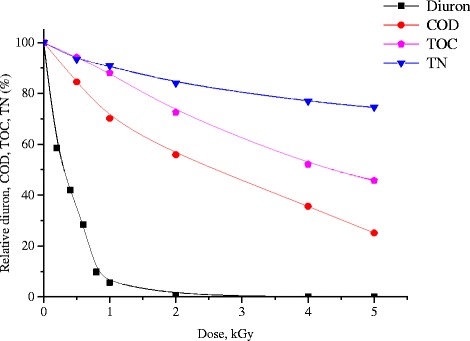
TOC, COD and TN results and removal of diuron. All of the experiments were carried out in 1.2 × 10^−4^ mol dm^−3^ diuron solutions before irradiation and after irradiating with various doses.

By measuring the changes of COD and TOC after irradiation, we monitored the rates of oxidation and mineralization. The TN measurements show whether the N content remains in the solution after degradation, or it leaves the solution in the form of N_2_. These measurements were conducted on samples saturated with air. COD and TOC decreased steadily with the absorbed dose (Figure 6), the decrease in COD was higher than in TOC. Partly oxidized molecules need less O_2_ for complete mineralization than the intact ones; however, their organic carbon content is similar to those of the starting molecules. For instance, in air saturated solutions at 1 kGy dose the decrease of COD and TOC was 30% and 7%, respectively. At this dose, about 95% of diuron was depleted. This result is similar to the one obtained by Zhang et al. [10] using 7.9 × 10^−5^ mol dm^−3^ diuron concentration; they found practically 100% removal of the starting molecules and 34 % TOC removal. TN decreased only slightly during the treatment showing that nitrogen mainly remained in the liquid phase.

The initial slope of the COD-dose plot is c.a. 11 mg dm^−3^ kGy^−1^, so at 1 kGy dose ~3.4 × 10^−4^ mol dm^−3^ O_2_ was built in the products. At this dose 2.8 × 10^−4^ mol dm^−3^^•^OH was introduced into the solution, the O_2_ molecules built-in/^•^OH introduced into the solution molar ratio is c.a. 1.2. When an O_2_ is incorporated in a product four-electron oxidation occurs. Therefore ^•^OH, the one-electron oxidant, induces more than four-electron oxidations.

## Discussion

Using the UV spectroscopic and LC-MS/MS results, the yields of diuron degradation are calculated to be in the 0.15 − 0.3 μmol J^−1^ range. The *G*-values of chloride release are between 0.07 − 0.15 μmol J^−1^ (two Cl-atom elimination). Based on the COD values measured in air saturated solution the O_2_/^•^OH ratio is c.a. 1.2. The value is much higher than the ratio established for fenuron, ~0.7 [11] and it is also higher than found for the majority of molecules studied so far in our laboratory (0.25 − 1.0 [17-19]). The high ratio in the present study indicates effective diuron degradation in air saturated solution.

**Scheme 1:**
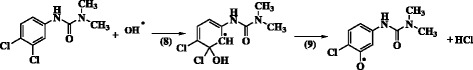
^•^OH attack to diuron at the *meta*-position.

^•^OH is expected to add to any of the carbon atoms of the aromatic ring. The addition at a carbon atom bearing the chlorine atom (8), may be followed by HCl elimination (9) as it was shown e.g. for diclofenac [20,21]. Canle Lopez et al. [5] suggest such chlorine release in photocatalytic oxidation of diuron, too. ^•^OH attack at the *meta*-position is shown in Scheme 1.

This reaction ultimately gives rise to phenoxyl type radical formation, the absorption bands in the transient spectrum at ~330 nm and at ~460 nm are probably due to this transient intermediate (Figure 1). The dechlorination yields in both N_2_O bubbled and air saturated solutions are smaller than the ^•^OH yields. Only a small fraction of ^•^OH reaction leads to dechlorination. Based on the higher dechlorination yield in aerated solution compared to N_2_O-saturated solution, one may think that the O_2_^−•^/HO_2_^•^ pair also induces dechlorination. O_2_^−•^ is a reductive radical with standard one-electron reduction potential of −0.33 V (*vs.* NHE) [22], while the standard one-electron reduction potential of diuron is calculated to be 1.72 V [23]. The dechlorination of chloroanilines in microbial experiments is demonstrated in several papers [24]: in the case of dichloroanilines first the *para-* and then the *meta-*chlorines are involved in the process. In our experiments carried out in the presence of Na formate, when O_2_^−•^ is the main reactive species, just a small dechlorination was found. Because of the low yield one may also think that the dechlorination is due to some side reaction in these solutions, e.g. small fraction of ^•^OH or e_aq_^−^ is not scavenged by HCO_2_^−^ or O_2_, respectively, and reacts with diuron inducing dechlorination. In the photocatalytic experiments of Canle Lopez et al. [5] a competition was found between the reactions of e^−^, transferred to the conduction band, with O_2_ and with diuron. Based on our experiments we may say that dissolved O_2_ in the solution promotes the Cl^−^ elimination from the adduct. Low reactivity of the O_2_^−•^/HO_2_^•^ pair with aromatic molecules was also reported in other works [18,25].

**Scheme 2:**
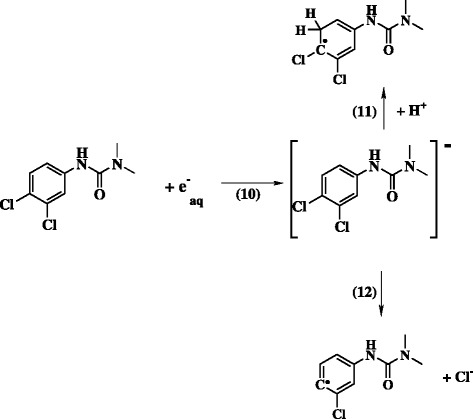
Reactions of e_aq_^−^ with diuron.

The rate coefficient of hydrated electron reaction with diuron is one order of magnitude higher than with fenuron due to the enhanced electron-withdrawing effect of the two Cl atoms attached to the aromatic ring. The electron is accommodated on the ring and the adduct undergoes reversible protonation with p*K*_a_ of 4.30 ± 0.04 [3]. The results in Figure 3 show that about 25% of the e_aq_^−^ reactions induce chloride release. The mechanism suggested for chloride release is shown in Scheme **2**, Reaction (12).

The phenyl radicals formed in (12) may be stabilized in bimolecular radical-radical or radical-molecule reactions. The expected products are molecules with one chlorine atom on the ring in *para*- (monuron) or in *meta*-position. These products were observed in our LC-MS/MS investigations, however, with quite low yields.

**Scheme 3:**
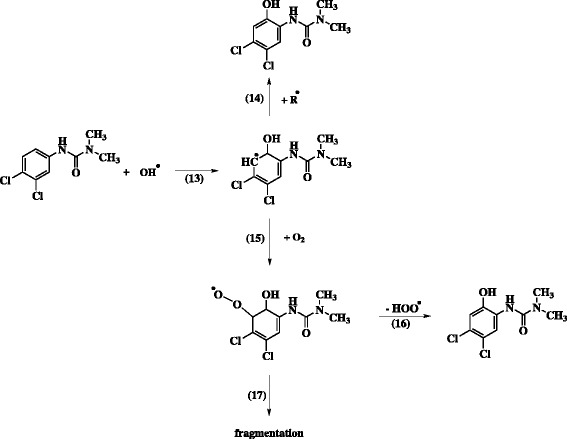
Reactions of ^•^OH with diuron in the presence and in the absence of O_2_.

In the present work and also in most of other AOP experiments, where ^•^OH played important role in the degradation [3-7], phenol type molecules were observed as important intermediate products. These phenols may form through the identified transient intermediates, hydroxycyclohexadienyl radicals shown in Scheme 3, Reaction (13).

In the absence of dissolved O_2_ the hydroxycyclohexadienyl radicals decay in bimolecular reaction with other radicals (R^•^) present: the reaction may yield phenol type molecules (Scheme 3, Reaction (14)). In the presence of dissolved O_2_ the transient radicals can transform to peroxy radicals (Reaction (15)). The peroxy radicals formed from hydroxycyclohexadienyl radicals may either eliminate HO_2_^•^ forming phenol compounds (Reaction (16)) or may undergo stepwise oxidation to open-chain carboxylic acids. These reaction pathways are well known for different aromatics [26,27]. The high degradation rate in air saturated solutions, as compared to reactions under other conditions, can be attributed to the involvement of oxygen in the process.

Products that reflect ^•^OH reaction with a methyl group were also observed in the presence of dissolved O_2_. However, based on the abundances it seems that ^•^OH predominantly reacts with the aromatic ring and not with the methyl groups.

## Conclusions

During irradiation of dilute diuron solutions effective degradation was observed, which was higher under oxidative than reductive conditions. Under oxidative circumstances the intermediate products observed were similar to those found in other Advanced Oxidation Processes: hydroxylation in the ring, hydroxylation coupled to chlorine release and products reflecting reactions on one of the methyl groups. The presence of dissolved oxygen enhanced the rate of degradation. As the COD values show, the one-electron-oxidant ^•^OH on average induces five-electron oxidations. The high oxidation rate is attributed to the reactions of the primarily formed radicals with dissolved O_2_ and the subsequent reactions of the peroxy radical.
